# Ratios of central venous-to-arterial carbon dioxide content or tension to arteriovenous oxygen content are better markers of global anaerobic metabolism than lactate in septic shock patients

**DOI:** 10.1186/s13613-016-0110-3

**Published:** 2016-02-03

**Authors:** Jihad Mallat, Malcolm Lemyze, Mehdi Meddour, Florent Pepy, Gaelle Gasan, Stephanie Barrailler, Emmanuelle Durville, Johanna Temime, Nicolas Vangrunderbeeck, Laurent Tronchon, Benoît Vallet, Didier Thevenin

**Affiliations:** Department of Anesthesiology and Critical Care Medicine, Service de Réanimation polyvalente, Centre Hospitalier du Dr. Schaffner, 99 route de La Bassée, 62307 Lens Cedex, France; Department of Anesthesiology and Critical Care Medicine, Centre Hospitalier Universitaire de Lille, Lille, France

**Keywords:** Anaerobic metabolism, Oxygen consumption, Venous-to-arterial carbon dioxide difference, Lactate, Venous oxygen saturation, Acute circulatory failure, Tissue hypoxia, Septic shock

## Abstract

**Background:**

To evaluate the ability of the central venous-to-arterial CO_2_ content and tension differences to arteriovenous oxygen content difference ratios (∆ContCO_2_/∆ContO_2_ and ∆PCO_2_/∆ContO_2_, respectively), blood lactate concentration, and central venous oxygen saturation (ScvO_2_) to detect the presence of global anaerobic metabolism through the increase in oxygen consumption (VO_2_) after an acute increase in oxygen supply (DO_2_) induced by volume expansion (VO_2_/DO_2_ dependence).

**Methods:**

We prospectively studied 98 critically ill mechanically ventilated patients in whom a fluid challenge was decided due to acute circulatory failure related to septic shock. Before and after volume expansion (500 mL of colloid solution), we measured cardiac index, VO_2_, DO_2_, ∆ContCO_2_/∆ContO_2_ and ∆PCO_2_/∆ContO_2_ ratios, lactate, and ScvO_2_. Fluid-responders were defined as a ≥15 % increase in cardiac index. Areas under the receiver operating characteristic curves (AUC) were determined for these variables.

**Results:**

Fifty-one patients were fluid-responders (52 %). DO_2_ increased significantly (31 ± 12 %) in these patients. An increase in VO_2_ ≥ 15 % (“VO_2_-responders”) concurrently occurred in 57 % of the 51 fluid-responders (45 ± 16 %). Compared with VO_2_-non-responders, VO_2_-responders were characterized by higher lactate levels and higher ∆ContCO_2_/∆ContO_2_ and ∆PCO_2_/∆ContO_2_ ratios. At baseline, lactate predicted a fluid-induced increase in VO_2_ ≥ 15 % with AUC of 0.745. Baseline ∆ContCO_2_/∆ContO_2_ and ∆PCO_2_/∆ContO_2_ ratios predicted an increase of VO_2_ ≥ 15 % with AUCs of 0.965 and 0.962, respectively. Baseline ScvO_2_ was not able to predict an increase of VO_2_ ≥ 15 % (AUC = 0.624).

**Conclusions:**

∆ContCO_2_/∆ContO_2_ and ∆PCO_2_/∆ContO_2_ ratios are more reliable markers of global anaerobic metabolism than lactate. ScvO_2_ failed to predict the presence of global tissue hypoxia.

**Electronic supplementary material:**

The online version of this article (doi:10.1186/s13613-016-0110-3) contains supplementary material, which is available to authorized users.

## Background

The aim of volume expansion, during acute circulatory failure, is to increase cardiac index (CI) and oxygen delivery (DO_2_) and to improve tissue oxygenation. Unrecognizable and untreated global tissue hypoxia is thought to contribute to the development of multiple organ failure or death. The usual indicators of global tissue hypoxia, such as blood lactate and venous oxygen saturation, are misleading. Increased lactate levels in sepsis are traditionally viewed as the result of activation of anaerobic glycolysis pathway due to inadequate oxygen delivery. According to such paradigms, hyperlactatemia signals tissue hypoxia and hypoperfusion [1, 2]. Nevertheless, in septic states, lactate concentration may increase through other mechanisms unrelated to tissue oxygen debt [3, 4]. Therefore, hyperlactatemia does not necessarily reflect anaerobic metabolism secondary to cellular hypoxia.

Measurement of mixed or central venous oxygen saturation (ScvO_2_) has been advocated in order to detect global tissue hypoxia [5]. ScvO_2_ reflects the balance between oxygen consumption and supply. A low ScvO_2_ represents a high oxygen extraction (OE) in order to maintain oxygen consumption (VO_2_) in spite of low DO_2_. However, a low ScvO_2_ does not necessarily indicate the presence of VO_2_/DO_2_ dependency. It is when ScvO_2_ cannot decrease proportionally to the decline of DO_2_ to maintain VO_2_, that cell moves from aerobic to anaerobic metabolism, leading to tissue hypoxia [6]. On the other hand, in septic shock, due to impairment of OE, normal/high ScvO_2_ values can also be observed in the presence of oxygen debt [5, 6]. Therefore, other markers are needed to indicate the presence of anaerobic metabolism in critically ill patients.

Considering the ratio of the venous-to-arterial CO_2_ tension difference (∆PCO_2_) over the arterial-to-venous oxygen content difference (∆ContO_2_) as a surrogate of the respiratory quotient (RQ), it has been suggested that this ratio can be used as a marker of global anaerobic metabolism in critically ill patients [7]. Recently, Monnet et al. [8] found that this ratio, calculated from central venous blood, predicted an increase in VO_2_ after a fluid-induced increase in DO_2_ and, thus, can be able to detect the presence of global tissue hypoxia. However, PCO_2_ is not equivalent to CO_2_ content, and the PCO_2_/CO_2_ content relationship is curvilinear rather than linear and is influenced by many factors such as pH and oxygen saturation (Haldane effect). Although VCO_2_/VO_2_ might be better reflected by the venous-to-arterial CO_2_ content over ∆ContO_2_ (∆ContCO_2_/∆ContO_2_) ratio, there is no report in the literature whether this latter ratio can predict more accurately a situation of anaerobic metabolism than ∆PCO_2_/∆ContO_2_ ratio. Therefore, the aim of our study was to evaluate the ability of ∆ContCO_2_/∆ContO_2_, ∆PCO_2_/∆ContO_2_, ScvO_2_, and blood lactate to predict the presence of the activation of global anaerobic metabolism through VO_2_/DO_2_ dependence in septic shock patients. The presence of VO_2_/DO_2_ dependency phenomenon was characterized by the increase in VO_2_ after an acute increase in DO_2_ induced by volume expansion (VE).

## Methods

This prospective single-center observational study was conducted in a general adult intensive care unit (ICU) after approval by our local institutional ethics committee (Lens Hospital, France). Informed consent was obtained from each subject’s next of kin.

### Patients

We studied mechanically ventilated patients for whom the attending physician decided to perform a VE due to the presence of at least one clinical sign of inadequate tissue perfusion due to septic shock [2]: (a) systolic arterial pressure <90 mmHg, mean arterial pressure <65 mmHg, or the need for vasopressor infusion; (b) skin mottling; (c) lactate level >2 mmo/L; or (d) urinary output <0.5 mL/kg/h for ≥2 h. Septic shock was defined according to international criteria [9]. Patients had also to be monitored by PiCCO device (PiCCO, Pulsion Medical System, Munich, Germany) as part of routine management of persistent signs of tissue hypoperfusion in our ICU. Exclusion criteria were: liver failure as defined by Sequential Organ Failure Assessment score, pregnancy, age <18 years old, moribund, and risk of fluid loading-induced pulmonary edema.

### Measurements

CI was obtained with the PiCCO monitor by triplicate central venous injections, in either the internal jugular or subclavian vein, of 20 mL of iced 0.9 % saline solution and recorded as the average of the three measurements.

Arterial lactate levels, arterial, and central venous blood gas were measured using the GEM Premier 4000 (Instrumentation Laboratory Co, Paris, France). The central venous blood was obtained from a central venous catheter with the tip confirmed to be in the superior vena cava at the entrance, or in the right atrium by radiograph. The ∆PCO_2_ was calculated as the difference between the central venous carbon dioxide tension and the arterial carbon dioxide tension. The arterial (CaO_2_) and central venous (CcvO_2_) oxygen contents were calculated using the standard formulas (Additional file 1: Supplementary material). The ∆ContO_2_ (mL) was calculated as CaO_2_–CcvO_2_. The DO_2_ was calculated by using the formula: DO_2_ (mL/min/m^2^) = CaO_2_ × CI × 10. The VO_2_ was calculated using the following formula: VO_2_ (mL/m^2^) = CI × ∆ContO_2_ × 10. Oxygen extraction was defined as: OE = VO_2_/DO_2_.

We also determined the central venous-to-arterial difference in blood CO_2_ content [∆ContCO_2_] according to Douglas et al. [10] (Additional file 1: Supplementary material).

### Study protocol

At baseline, a first set of measurements was performed, including hemodynamic and tissue oxygenation variables (Additional file 1: Supplementary material), arterial lactate level, ∆PCO_2_, ∆ContCO_2_/∆ContO_2_ ratio, and ∆PCO_2_/∆ContO_2_ ratio. A 500 mL of colloid solution (4 % human serum albumin, Vialebex^®^; LFB) was infused to the patient over 15 min via a specific venous line. Immediately after VE, a second set of measurement was recorded, including the same hemodynamic and tissue oxygenation variables. Ventilation parameters and doses of norepinephrine, dobutamine, and sedation drugs were kept constant during the VE.

### Statistical analysis

According to changes in CI after the 500-mL VE, patients were classified as fluid-responders (≥15 % increase in CI) or fluid-non-responders. Also, in the fluid-responders’ group, patients were separated into two subgroups according to their increase in VO_2_ (< or ≥15 %) induced by VE [8]. All data are expressed as mean ± SD when they are normally distributed, or as median [25–75 %, interquartile range, (IQR)] when they are non-normally distributed. The normality of data distribution was assessed using the Kolmogorov–Smirnov test. Comparisons of values between different groups of patients were made by two-tailed Student’s t test, or Mann–Whitney U test, as appropriate. Pairwise comparisons between different study times were assessed using paired Student’s t test or Wilcoxon’s test, as appropriate. Linear correlations were tested by using the Pearson or the Spearman test, as appropriate. To adjust for the regression to the mean phenomenon, the absolute change in variables over time was also analyzed by performing an analysis of covariance (ANCOVA) with the absolute change in variable as dependent variable, the group of patients as a factor, and the baseline value as a covariate.

Receiver operating characteristics (ROC) curves were constructed to evaluate the ability of tissue oxygenation variables at baseline to predict an increase of VO_2_ ≥ 15 % after VE in fluid-responders’ group. The areas under the ROC curves (AUCs) were compared using the nonparametric technique described by DeLong et al. [11]. The best cutoff of a ROC curve was chosen with the highest Youden index [12]. Usually, variables are considered of good clinical tool (having good discriminative properties tests) when the inferior limits of the 95 % confidence interval (CI) of their AUC are more than 0.75 [12]. For this purpose, considering a proportion of VO_2_ responders of 21 % [8], 51 fluid-responder’s patients are required for a power of 90 % and an alpha risk of 0.05. Assuming a proportion of responders close to 50 %, about 102 patients would be necessary. Statistical analysis was performed using STATA 14.0 (StataCorp LP, College Station, Texas, USA) and SPSS for Windows release 17.0 (Chicago, Illinois, USA). p < 0.05 was considered statistically significant. All reported P values are two-sided.

## Results

### Patients

Ninety-eight septic shock patients were prospectively included in this study. The main characteristics of the cohort are summarized in Table 1. The median time between the start of care and enrollment was 1.7 [1.0–2.0] h. The major source of infection was pneumonia (55 %) with ICU mortality rate of 42.8 %.

**Table 1:** Baseline characteristics of the patients (n = 98)

Age (mean ± SD, years)	63 ± 11
Body masse index (mean ± SD, kg/m^2^)	26.6 ± 5.4
Gender (female/male) (n)	41/57
Simplified acute physiologic score (mean ± SD)	64 ± 18
Sequential organ failure assessment score (median [IQR])	10 [6–12]
ICU mortality [n (%)]	42 (42.8)
Time between diagnosis and inclusion (median [IQR], h)	1.7 [1.0–2.0]
Infection source [n (%)]	
Pneumonia	54 (55)
Peritonitis	34 (35)
Meningitis	5 (5)
Urinary tract infection	5 (5)
Patients receiving norepinephrine [n (%)]	98 (100)
Norepinephrine dose (median [IQR], µg/kg/min)	0.24 [0.11–0.55]
Patients receiving dobutamine [n (%)]	21 (21.4)
Dobutamine dose (median [IQR], µg/kg/min)	5 [5–10]
Mechanical ventilation [n (%)]	98 (100)

*IQR* interquartile range, *ICU* intensive care unit

### The whole population

At baseline, there was a significant but weak correlation between lactate and ∆PCO_2_/∆ContO_2_ (r = 0.33, p = 0.001) and between lactate and ∆ContCO_2_/∆ContO_2_ (r = 0.32, p = 0.001). Furthermore, fluid-induced changes in ∆PCO_2_/∆ContO_2_, ∆ContCO_2_/∆ContO_2_, and lactate were weakly correlated (r = 0.25, p = 0.01 and r = 0.21, p = 0.04; respectively). We found a good correlation between ∆ContCO_2_/∆ContO_2_ and ∆PCO_2_/∆ContO_2_ ratios (r = 0.63, p < 0.001). Fluid-induced changes in ∆PCO_2_/∆ContO_2_ and ∆ContCO_2_/∆ContO_2_ ratios were also well related (r = 0.63, p < 0.001).

### Effects of volume expansion on hemodynamic variables

VE increased CI by more than 15 % in 51 patients who were “fluid-responders” (52 %; Additional file 2: Table S1). In these patients, VE significantly increased CI by 30 ± 13 %, DO_2_ by 31 ± 12 %, and VO_2_ by 25 ± 26 % (Additional file 2: Table S1). Volume expansion induced less than 15 % increase in CI in 47 patients (48 %). In these patients, we observed no significant changes in DO_2_ and VO_2_.

### Differences between VO_2_-responders and VO_2_-non-responders in fluid-responders’ group

**Fig. 1:**
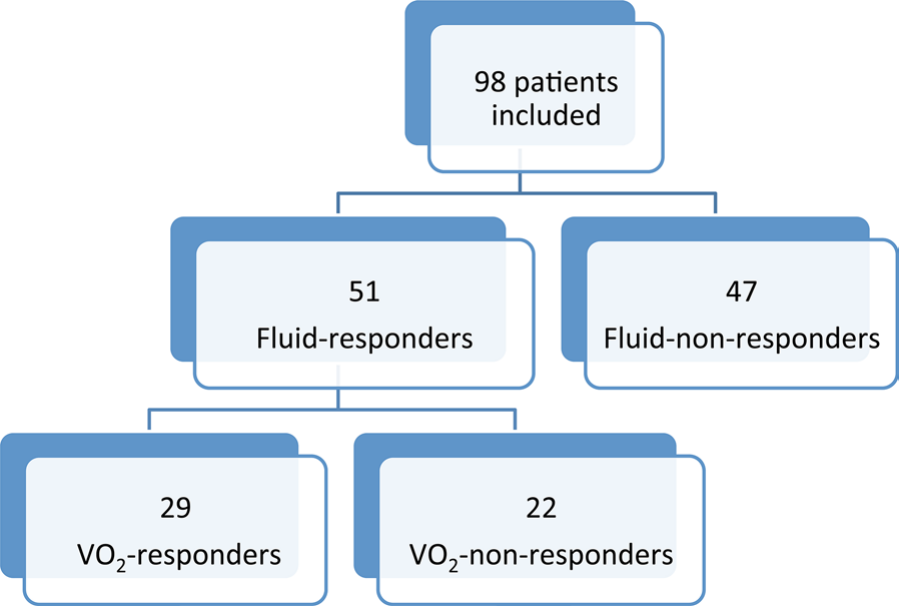
Flow chart showing the original 98 patients separated according to their response to volume expansion in terms of cardiac index, fluid responsiveness, and oxygen consumption (VO_2_)

Of the 51 fluid-responders, VE increased VO_2_ ≥ 15 % (45 ± 16 %) in 29 who were “VO_2_-responders.” In the remaining 22 fluid-responders, VE did not significantly change VO_2_ “VO_2_-non-responders” (Table 2; Fig. 1).

**Table 2:** Hemodynamic and tissue oxygenation parameters before and after 500 mL of volume expansion according to the response of VO_2_ in fluid-responder patients

	VO_2_ change ≥15 % (n = 29)		VO_2_ change <15 % (n = 22)	
	Before volume expansion	After volume expansion	Before volume expansion	After volume expansion
Heart rate (beats/min)	108 ± 34	102 ± 26*	110 ± 27	109 ± 26
Mean arterial pressure (mmHg)	66 ± 16	79 ± 13*	74 ± 13	85 ± 10*
Cardiac index (L/min/m^2^)	2.59 [1.42–3.03]	3.02 [2.09–3.71]*	3.25 [2.49–3.65]^#^	3.81 [3.48–4.23]^#,^*
Stroke index (mL/m^2^)	19.4 [13.1–35.9]	27.0 [22.5–43.8]*	30.8 [21.5–34.1]^#^	41.0 [31.6–41.4]*
Minute ventilation (L/min)	10.4 ± 2.0	–	8.5 ± 1.5^#^	–
Arterial pH	7.35 [7.32–7.38]	7.36 [7.35–7.37]	7.23 [7.21–7.30]^#^	7.22 [7.22–7.31]^#^
Base excess (mmol/L)	–7.21 ± 4.74	–7.11 ± 4.21	–8.15 ± 4.20	–8.25 ± 3.89
SaO_2_ (%)	99 [90–100]	98 [94–100]	94 [91–96]^#^	95 [92–98]^#,^*
Hemoglobin (g/dL)	10.1 ± 1.3	9.5 ± 1.1*	10.7 ± 1.0	10.1 ± 0.9^#,^*
CaO_2_ (mL)	12.8 ± 2.0	12.8 ± 1.7	13.2 ± 1.4	13.2 ± 1.2
PaCO_2_	31 ± 5	31 ± 6	43 ± 8^#^	42 ± 7^#^
CaCO_2_ (mL)	37.1 [30.0–49.2]	37.2 [28.8–46.4]*	40.5 [35.6–46.9]	37.2 [34.9–42.8]*
Venous pH	7.31 [7.24–7.34]	7.33 [7.27–7.33]	7.22 [7.18–7.24]^#^	7.21 [7.20–7.27]^#,^*
ScvO_2_ (%)	63 ± 11	63 ± 10	58 ± 14	69 ± 10^#^,*
CcvO_2_ (mL)	8.4 ± 2.0	8.1 ± 1.6*	8.2 ± 2.2	9.6 ± 1.8^#,^*
PcvCO_2_ (mmHg)	40 ± 7	38 ± 7*	49 ± 7^#^	47 ± 6^#,^*
CcvCO_2_ (mL)	43.4 [35.1–53.3]	41.4 [31.8–49.6]*	43.6 [40.2–47.3]	39.9 [37.9–43.3]*
DO_2_ (mL/min/m^2^)	297 ± 86	381 ± 84*	409 ± 80^#^	529 ± 84^#,^*
VO_2_ (mL/min/m^2^)	95 ± 27	134 ± 30*	148 ± 43^#^	145 ± 37
OE	0.34 ± 0.11	0.37 ± 0.10*	0.37 ± 0.15	0.28 ± 0.09^#,^*
∆PCO_2_ (mmHg)	7.0 [6.0–14.0]	6.0 [5.0–12.0]*	4.0 [3.7–9.0]^#^	3.0 [3.0–6.0]^#,^*
∆ContO_2_ (mL)	4.3 ± 1.6	4.7 ± 1.4*	5.0 ± 2.1	3.7 ± 1.1^#,^*
∆ContCO_2_ (mL)	5.2 [3.9–6.3]	3.0 [2.8–4.2]*	1.8 [0.3–4.7]^#^	1.5 [0.5–3.3]^#,^*
∆PCO_2_/∆ContO_2_ (mmHg/mL)	2.20 [1.70–2.33]	1.66 [1.10–1.96]*	1.05 [1.02–1.14]^#^	1.13 [0.83–1.35]^#^
∆ContCO_2_/∆ContO_2_	1.22 [1.05–1.26]	0.65 [0.65–0.70]*	0.53 [0.09–0.64]^#^	0.46 [0.14–0.70]^#^
Lactate (mmol/L)	5.8 [3.2–7.6]	5.0 [2.8–6.9]*	2.9 [2.1–4.9]^#^	2.7 [1.4–4.4]^#,^*

Data are expressed as mean ± SD or as median [interquartile range, 25–75]

*SaO*_*2*_ arterial oxygen saturation, *CaO*_*2*_ arterial oxygen content, *PaCO*_*2*_ arterial carbon dioxide tension, *ScvO*_*2*_ central venous oxygen saturation, *CcvO*_*2*_ central venous oxygen content, *PcvCO*_*2*_ central venous carbon dioxide tension, *DO*_*2*_ oxygen delivery, *VO*_*2*_ oxygen consumption, *∆PCO*_*2*_ venous–arterial carbon dioxide tension difference, *CaCO*_*2*_ arterial carbon dioxide content, *CcvCO*_*2*_ central venous carbon dioxide content, *∆ContO*_*2*_ arterial-to-central venous oxygen content difference, *∆ContCO*_*2*_ central venous-to-arterial carbon dioxide content difference, *OE* oxygen extraction

* p < 0.05 after vs. before volume expansion. ^#^p < 0.05 patients with a VO_2_ < 15 % versus patients with a VO_2_ ≥ 15 %

At baseline, compared with the VO_2_-non-responders, lactate levels and ∆PCO_2_/∆ContO_2_ and ∆ContCO_2_/∆ContO_2_ ratios were significantly higher in VO_2_-responders, whereas ScvO_2_ value was not significantly different between these two subgroups (Table 2). In these patients, ∆PCO_2_/∆ContO_2_ and ∆ContCO_2_/∆ContO_2_ ratios decreased significantly by 30 ± 13 and 45 ± 24 % (respectively) with VE, whereas they did not change in VO_2_-non-responders (Table 2). ScvO_2_ significantly increased (22 ± 16 %) with VE only in VO_2_-non-responders (Table 2).

Lactate levels decreased significantly with VE in both VO_2_-responders and VO_2_-non-responders. The magnitude of decrease in lactate levels was not different between these two subgroups (13 ± 7 % for VO_2_-responders vs. 13 ± 10 % for VO_2_-non-responders, p = 0.98, Table 2).

Among fluid-responder patients, VE-induced changes in ∆PCO_2_/∆ContO_2_, ∆ContCO_2_/∆ContO_2_, and ScvO_2_, but not in lactate, were significantly higher, after adjustment to their baseline values, in VO_2_-responders than in VO_2_-non-responders (ANCOVA, p < 0.001, p = 0.006, p < 0.001, and p = 0.18; respectively).

VE-induced change in VO_2_ was not significantly correlated with the changes in lactate (r = −0.17, p = 0.22). However, changes in both ∆PCO_2_/∆ContO_2_ and ∆ContCO_2_/∆ContO_2_ ratios induced by VE were well correlated with changes in VO_2_ (r = −0.52, p < 0.001, and r = −0.59, p < 0.001; respectively).

### Ability of tissue oxygenation variables to predict the response of VO_2_ to VE in fluid-responders

**Fig. 2:**
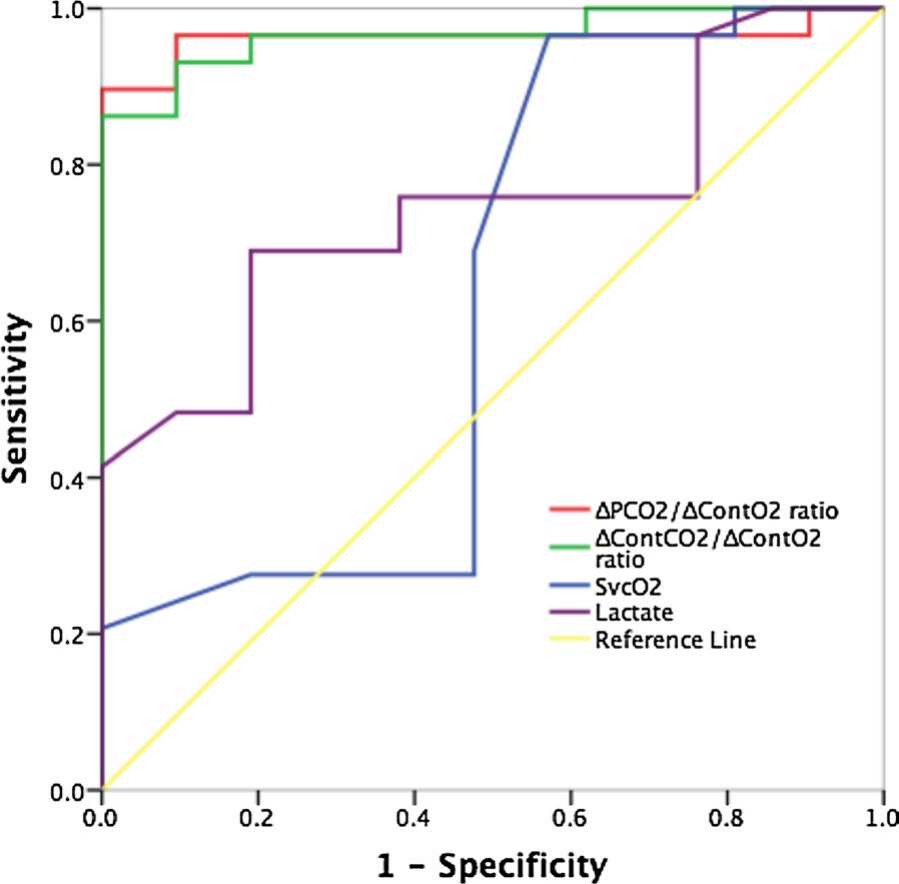
Receiver operating characteristic (ROC) curves showing the ability of baseline ∆ContCO_2_/∆ContO_2_, ∆PCO_2_/∆ContO_2_, lactate, and ScvO_2_ to predict an increase in oxygen consumption of ≥15 % induced by volume expansion

The ability of ∆PCO_2_/∆ContO_2_ and ∆ContCO_2_/∆ContO_2_ at baseline to predict an increase of VO_2_ ≥ 15 % induced by VE was excellent in fluid-responder patients with AUCs of 0.962 (95 % CI 0.900–1.000) and 0.965 (95 % CI 0.918–1.000), respectively (Fig. 2). The AUC for baseline value of lactate was 0.745 [(95 % CI 0.608–0.883), p = 0.003 vs. 0.5]. However, ScvO_2_ at baseline did not predict the VO_2_ increase of ≥15 % [AUC = 0.624, (95 % CI 0.449–0.798), p = 0.14 vs. 0.5].

AUCs for baseline ∆PCO_2_/∆ContO_2_ and ∆ContCO_2_/∆ContO_2_ ratios were both significantly larger than the AUCs for baseline lactate (p = 0.003 and p = 0.002, respectively) and baseline ScvO_2_ (p < 0.001 for both ratios, Fig. 2). However, there were no significant differences between AUC for ∆PCO_2_/∆ContO_2_ and AUC for ∆ContCO_2_/∆ContO_2_ (p = 0.80), neither between AUC for baseline lactate and AUC for baseline ScvO_2_ (p = 0.24).

The best cutoff values at baseline, when predicting VO_2_ responsiveness, were ≥1.68 mmHg/mL for ∆PCO_2_/∆ContO_2_ ratio [sensitivity = 90 % (95 % CI 71–97 %); specificity = 100 % (95 %CI 81–100 %)], ≥1.02 for ∆ContCO_2_/∆ContO_2_ ratio [sensitivity = 86 % (95 % CI 67–95 %); specificity = 100 % (95 % CI 81–100 %)], and ≥4.6 mmol/L for lactate [sensitivity = 69 % (95 % CI 49–84 %); specificity = 77 % (95 % CI 54–91 %)] (Table 3). Even though the AUC for baseline ScvO_2_ was not significantly different from 0.5, a ScvO_2_ value ≥80 % had a specificity of 100 % (95 % CI 81–100 %) but a sensitivity of 21 % (95 % CI 9–40 %) to predict the VO_2_ increase ≥15 %. Thus, in fluid-responders, a ScvO_2_ value ≥80 % was always associated with an increase in VO_2_ ≥ 15 % induced by VE. Compared with patients with a baseline ScvO_2_ < 80 %, these patients had higher baseline lactate levels [4.3 (2.3–6.3) mmol/L vs. 7.6 (5.6–8.1) mmol/L, p = 0.011; respectively], higher baseline ∆PCO_2_/∆ContO_2_ ratio [1.46 (1.05–2.17) mmHg/mL vs. 2.20 (2.20–2.38) mmHg/mL, p = 0.011; respectively], and higher baseline ∆ContCO_2_/∆ContO_2_ ratio [0.94 (0.53–1.05) vs. 1.52 (1.42–1.62), p < 0.001; respectively].

**Table 3:** Diagnostic ability of baseline values of central venous-to-arterial CO_2_ tension and content differences to arterial-to-venous oxygen content difference ratios, and baseline lactate values to predict fluid-induced increase in oxygen consumption ≥15 % in fluid-responder patients (n = 51)

	Best cutoff value	Se (%) (95 % CI)	Sp (%) (95 % CI)	PPV (%) (95 % CI)	NPV (%) (95 % CI)	LR^+^ (95 % CI)	LR^−^ (95 % CI)
∆PCO_2_/∆ContO_2_ (mmHg/mL)	1.68	90 (71–97)	100 (81–100)	100 (84–100)	88 (68–97)	Infinity	0.10 (0.03–0.30)
∆ContCO_2_/∆ContO_2_	1.02	86 (67–95)	100 (81–100)	100 (83–100)	85 (64–95)	Infinity	0.14 (0.05–0.34)
Lactate (mmol/L)	4.6	69 (49–84)	77 (54–91)	80 (59–92)	65 (44–82)	3.03 (1.35–6.81)	0.40 (0.23–0.71)

*Se* sensitivity, *Sp* specificity, *PPV* positive predictive value, *NPV* negative predictive value, *LR* ^*+*^ positive likelihood ratio, *LR* ^*−*^ negative likelihood ratio, *CI* confidence interval

### Comparisons between patients with ScvO_2_ > 70 % and patients with ScvO_2_ ≤ 70 % at baseline in the whole population

Only 28 patients had a ScvO_2_ of more than 70 % at baseline (Additional file 3: Table S2). They had significantly lower baseline VO_2_ and higher baseline DO_2_ compared with patients with low baseline ScvO_2_. The mean baseline value of VO_2_ significantly increased with volume expansion only in patients with ScvO_2_ > 70 % (Additional file 3: Table S2).

## Discussion

The main findings of our study were that in fluid-responder septic shock patients (1) the ability of ∆ContCO_2_/∆ContO_2_ and ∆PCO_2_/∆ContO_2_ ratios to predict the presence of global anaerobic metabolism was excellent and higher than lactate; (2) ∆ContCO_2_/∆ContO_2_ ratio was not a better marker of global anaerobic metabolism than ∆PCO_2_/∆ContO_2_ ratio; (3) ScvO_2_ failed to detect the presence of global tissue hypoxia, except for values ≥80 %.

When DO_2_ is acutely reduced, VO_2_ remains stable (oxygen supply independency) because the tissues adapt their OE proportionally. When DO_2_ falls below a critical low value, a proportionate increase in OE cannot be maintained and the VO_2_ starts to fall (oxygen supply dependency) and tissue hypoxia occurs as reflected by an abrupt increase in blood lactate concentration [13–15]. Thus, VO_2_/DO_2_ dependence has been considered to be a hallmark of tissue hypoxia and the activation of anaerobic metabolism [14–16], although it has been challenged because of the methodological limitations (mathematical coupling) in the VO_2_/DO_2_ relationship assessment [17, 18].

We defined an increase in VO_2_ ≥ 15 % as a clinically significant augmentation by similarity to the definition of the increase in cardiac index, since VO_2_ is proportional to this variable. This cutoff value was chosen by the fact that the least significant change in CI measured by transpulmonary thermodilution is 11.0 % when 20 mL is used to perform iced saline injections in triplicate (unpublished data). On the other hand, this definition of “VO_2_ response” allows us comparing our findings with those of a previous study [8]. We confirm the results of Monnet et al. [8] that calculating VO_2_ from the central instead of the mixed venous blood also allows to detect the presence of anaerobic metabolism through VO_2_/DO_2_ dependence. Indeed, in our study, the baseline lactate concentration was elevated in patients with VO_2_/DO_2_ dependency phenomenon and higher than in patients with VO_2_/DO_2_ independency (Table 2). Moreover, it is hard to believe that mathematical coupling of measurement errors was responsible for the VO_2_/DO_2_ dependency in our study. Indeed, we observed that VO_2_/DO_2_ dependency occurred in one subgroup of patients but not in others, despite similar changes in DO_2_ (Table 2). Such a methodological problem can hardly account for the existence of VO_2_/DO_2_ dependency only in one subgroup. If that were an issue, one would expect it to influence results uniformly. Finally, the increase in VO_2_ could have resulted from an additional non-mitochondrial non-oxidative oxygen uptake when dysoxia has resolved [19]. However, this mechanism is less likely to have occurred in our study because the observed mean slope of the VO_2_/DO_2_ relationship in the subgroup of VO_2_-responders was 47.8 ± 10 %, suggesting VO_2_/DO_2_ dependency and activation of anaerobic metabolism (Table 2) [20].

In experimental conditions of tissue hypoxia, a smaller reduction in VCO_2_ than VO_2_ has been observed, suggesting a non-aerobic production of CO_2_ [21–23]. Therefore, the occurrence of a high RQ may be considered as a sign of anaerobic metabolism. Recently, Monnet et al. [8] have used the ∆PCO_2_/∆ContO_2_ ratio as a surrogate of RQ and found that a ∆PCO_2_/∆ContO_2_ ratio at baseline ≥ 1.8 mmHg/mL predicted accurately VO_2_/DO_2_ dependence among patients whose DO_2_ increased after fluid administration. Our results agree with those findings, and interestingly, the observed cutoff value of this ratio, in our septic shock patients, was almost similar to what was found in the Monnet et al. report [8].

The use of ∆PCO_2_/∆ContO_2_ ratio as a surrogate of VCO_2_/VO_2_ assumes that the PCO_2_/CO_2_ content relationship is quasi-linear, which may be true over the physiologic range of PCO_2_ [24]. However, this relationship can be affected by the degree of metabolic acidosis, hematocrit, and oxygen saturation (Haldane effect), and it becomes nonlinear if these factors change [25]. In this regard, it has been shown that venous-to-arterial PCO_2_ differences and venous-to-arterial CO_2_ content differences might change in opposite direction in splanchnic region under conditions of very low venous oxygen saturation [26]. Recently, Ospina-Tascon et al. found a significant association with mortality, in septic shock patients, for the mixed ∆ContCO_2_/mixed ∆ContO_2_ ratio but not for the mixed ∆PCO_2_/mixed ∆ContO_2_ ratio [27]. Thus, ∆ContCO_2_/∆ContO_2_ ratio could be a more reliable marker of tissue hypoxia than ∆PCO_2_/∆ContO_2_. However, we found that ∆ContCO_2_/∆ContO_2_ was not better predictor of tissue hypoxia than the ∆PCO_2_/∆ContO_2_ in septic shock patients (Fig. 2). It does not seem that Haldane effect has played an important role in our study. Furthermore, the degree of metabolic acidosis, as reflected by base excess, was not severe enough to significantly affect the PCO_2_/CO_2_ content relationship in our septic shock patients. Even though the ∆ContCO_2_/∆ContO_2_ ratio more physiologically mirrors RQ compared with ∆PCO_2_/∆ContO_2_, we found that both ratios can be used accurately to predict fluid responsiveness at tissue level. However, the computation of ∆PCO_2_/∆ContO_2_ ratio is less cumbersome and less subject to the risk of errors, and therefore, it is much easier to be used at the bedside.

Lactate value was not good to detect the presence of anaerobic metabolism in our septic shock patients. Our results are in discrepancy with previous findings [7, 8, 28]. However, hyperlactatemia does not necessarily reflect anaerobic metabolism secondary to tissue hypoxia, especially in septic states [3, 4, 29]. Other non-hypoxic mechanisms such as accelerated aerobic glycolysis induced by sepsis-associated inflammation [30], inhibition of pyruvate dehydrogenase [31], and impaired lactate clearance [32] may contribute to hyperlactatemia found in septic patients. In endotoxic states, lactate levels failed to discriminate between hypoxia and aerobiosis [33]. Furthermore, Rimachi et al. [34] found the presence of hyperlactatemia in 65 % of septic shock patients, but only 76 % of these patients also had a high lactate/pyruvate ratio confirming the non-hypoxic cause of hyperlactatemia in septic states. Moreover, in fluid-responder patients, we found no significant relationship between changes in VO_2_ induced by VE and changes in lactate levels, whereas changes in ∆PCO_2_/∆ContO_2_ and ∆ContCO_2_/∆ContO_2_ ratios were correlated well with changes in VO_2_. This finding suggests that these ratios respond to changes in global tissue oxygenation faster than blood lactate concentration likely due to the alteration of lactate clearance.

The majority (71 %) of our septic shock patients had a ScvO_2_ value ≤70 % at their inclusion in the study. This finding is due to the fact that patients, in our study, were recruited in the very early period of acute circulatory failure; the time between the start of care and enrollment was only 102 min. Within this period, septic shock patients are not fully resuscitated yet, and as a consequence, low values of ScvO_2_ are observed more frequently [35–37]. Even though our population seems to be different from that in the study of Monnet et al. [8], we confirm that ScvO_2_ is a poor predictor of the presence of anaerobic metabolism. This can be explained by the fact that ScvO_2_ is not a regulated parameter but an adaptive one that depends on four regulated constituents: oxygen consumption, hemoglobin, SaO_2_, and cardiac output. Therefore, ScvO_2_ is widely fluctuating. However, these results should not dissuade us from monitoring ScvO_2_ but encourage us to include it in a multimodal approach. Indeed, a low ScvO_2_ value reflects the inadequacy of oxygen supply, and fluid administration can be helpful in order to correct oxygen supply/demand imbalance, even in situations of VO_2_/DO_2_ independency, to avoid further decreases in DO_2_ below a critical value leading to tissue hypoxia. On the other hand, only ScvO_2_ values ≥80 % were able to predict the presence of global tissue hypoxia with a high specificity. All these patients also had higher lactate levels and higher ∆PCO_2_/∆ContO_2_ and ∆ContCO_2_/∆ContO_2_ ratios. This suggests that these patients had a greater alteration of their microcirculation due to sepsis than the other fluid-responder patients. However, this finding should be interpreted with caution, since only six patients had a baseline ScvO_2_ value ≥80 % in fluid-responders’ group, and our study was not designed for testing this hypothesis.

Contrary to what was found previously [8], DO_2_ did not decrease during VE, in fluid-non-responder patients, even though arterial hemoglobin significantly decreased by 5.6 ± 4.6 % (Additional file 2: Table S1), which was lower than that in the Monnet et al. study (8 ± 4 %) [8]. The discrepancy between the two studies may be due to dissimilar populations of patients and to the differences in the time to inclusion, which was longer in the study by Monnet et al. [8] than that in our study (6.1 vs. 1.7 h) explaining the more pronounced hemodilution effect in their study.

We believe our findings add significant values to the Monnet et al. study [8]. Indeed, we have demonstrated that the ∆PCO_2_/∆ContO_2_ ratio is a reliable marker of global anaerobic metabolism in the very early period of septic shock where patients are still not fully resuscitated and that ∆ContCO_2_/∆ContO_2_ is not superior to ∆PCO_2_/∆ContO_2_ for predicting the presence of global tissue hypoxia in these patients. Furthermore, our study shed the light on the fact that hyperlactatemia should not always be regarded as reflecting the presence of global tissue hypoxia, especially in septic shock patients. This finding is of clinical importance since elevated lactate values could incite the clinician to undertake unnecessary interventions, with their potentially harmful effects, such as tissue edema and positive fluid balance, which have constantly been associated with poorer outcome [38].

Our study presents several limitations. First, we used central venous blood instead of mixed venous to assess VO_2_- and CO_2_-derived variables, and thus, we might have missed the evaluation of the splanchnic oxygenation. However, our study is the second one that confirms that calculating oxygen-derived variables from the central venous blood is able to detect the presence of tissue hypoxia through VO_2_/DO_2_ dependence. Second, regional or local tissue hypoxia might not be detected by the assessment of the changes in global oxygen consumption. Finally, it is a single-center study, which may limit its external validity.

## Conclusion

∆ContCO_2_/∆ContO_2_ and ∆PCO_2_/∆ContO_2_ are excellent and better markers of global anaerobic metabolism than lactate in fluid-responder septic shock patients. Also, these parameters respond to changes in global oxygenation faster than lactate. ScvO_2_ cannot predict the presence of global tissue hypoxia, except for values ≥80 %.

## Additional files


10.1186/s13613-016-0110-3 Supplementary material.

10.1186/s13613-016-0110-3 Hemodynamic and tissue oxygenation parameters before and after 500 mL of volume expansion in responders and non-responders.

10.1186/s13613-016-0110-3 Hemodynamic and tissue oxygenation parameters before and after 500 mL of volume expansion according to the baseline ScvO_2_ groups.

## Abbreviations

CI: cardiac index
DO_2_: oxygen delivery
VO_2_: oxygen consumption
ScvO_2_: central venous oxygen saturation
∆PCO_2_: central venous-to-arterial carbon dioxide tension difference
∆ContCO_2_: central venous-to-arterial carbon dioxide content difference
∆ContO_2_: arterial-to-central venous oxygen content difference
CaO_2_: arterial oxygen content
CcvO_2_: central venous oxygen content
OE: oxygen extraction
RQ: respiratory quotient
VE: volume expansion
AUC: area under receiver operating characteristic curve
95 % CI: 95 % confidence interval
