# Supplementary material for: Ratios of central venous-to-arterial carbon dioxide content or tension to arteriovenous oxygen content are better markers of global anaerobic metabolism than lactate in septic shock patients
Source: Ann Intensive Care. 2016 Feb 3;6:10. doi: 10.1186/s13613-016-0110-3 (PMC4740480; doi:10.1186/s13613-016-0110-3)
Supplement: Supplementary file 1 — 10.1186/s13613-016-0110-3 Supplementary material. [file 13613_2016_110_MOESM1_ESM.doc]

**Ratios of central venous-to-arterial carbon dioxide content or tension to arteriovenous oxygen content are better markers of global anaerobic metabolism than lactate in septic shock patients**

Jihad MALLAT^1^ M.D., Malcolm LEMYZE^1^ M.D., Mehdi MEDDOUR^1^ M.D., Florent PEPY^1^ M.D., Gaelle GASAN^1^ M.D., Stephanie BARRAILLER^1^ M.D., Emmanuelle DURVILLE^1^ M.D., Johanna TEMIME^1^ M.D., Nicolas VANGRUNDERBEECK^1^ M.D., Laurent TRONCHON^1^ M.D., Benoît VALLET^2^ M.D., Ph.D., and Didier THEVENIN^1^ M.D.

**Supplementary Material**

**^1^** Department of Anesthesiology and Critical Care Medicine, Centre Hospitalier du Dr. Schaffner de Lens, France.

^2^ Department of Anesthesiology and Critical Care Medicine, Centre Hospitalier Universitaire de Lille, France.

**Corresponding Author:**

Jihad Mallat, MD

Centre Hospitalier du Dr. Schaffner

Service de Réanimation polyvalente

99 route de La Bassée, 62307 Lens cedex, France.

Phone : +33321691088

Fax : +33321691839

Email : mallatjihad@gmail.com

**Materials and Methods**

**Measurements**

Demographic data, acute circulatory failure etiology, the Simplified Acute Physiology Score (SAPS) II, and the Sequential Organ Failure Assessment (SOFA) scores were obtained on the day of enrollment.

Cardiac index (CI) was obtained with the PiCCO monitor by triplicate central venous injections, in either the internal jugular or subclavian vein, of 20 ml of iced 0.9 % saline solution and recorded as the average of the three measurements. We also recorded heart rate and systemic arterial pressures.

Arterial lactate levels, arterial and central venous blood gas were measured using the GEM Premier 4000 (Instrumentation Laboratory Co, Paris, France). The central venous blood was obtained from a central venous catheter with the tip confirmed to be in the superior vena cava at the entrance, or in, the right atrium by radiograph. The central venous-to-arterial carbonic dioxide tension difference (∆PCO_2_) was calculated as the difference between the central venous carbon dioxide tension (PcvCO_2_) and the arterial carbon dioxide tension (PaCO_2_). The arterial oxygen content was calculated as CaO_2_ (ml) = 1.34 × Hb (g/dl) × SaO_2_ + 0.003 × PaO_2_ (mmHg), where SaO_2_ is the oxygen saturation of arterial blood, Hb the hemoglobin concentration, and PaO_2_ the arterial oxygen tension. The central venous oxygen tension was calculated as CcvO_2_ (ml) = 1.34 × Hb (g/dl) × ScvO_2_ + 0.003 × PcvO_2_ (mmHg), where PcvO_2_ is the central venous oxygen tension, and ScvO_2_ is the central venous oxygen saturation. The C(a-cv)O_2_ (ml) was calculated as CaO_2_ − CcvO_2_. The DO_2_ was calculated by using the formula: DO_2_ (ml/min/m^2^) = CaO_2_ × CI × 10. The VO_2_ was calculated using the following formula: VO_2_ ((ml/m^2^) = CI × C(a-cv)O_2_  × 10. Oxygen extraction was defined as: OE= VO_2_/DO_2_.

We also determined the central venous-arterial difference in blood CO_2_ content [∆ContCO_2_] according to Douglas et al. [1]:

Blood CO_2_ content (ml) =

Plasma CCO_2_ × [1 – 0.0289 × (Hb)**/**(3.352 – 0.456 × SO_2_) × (8.142 – pH)]

where plasma CCO_2_ = 2.226 × S × plasma PCO_2_ × (1 + 10^pH – pK’^), CCO_2_ is CO_2_ content, SO_2_ is oxygen saturation, S is the plasma CO_2_ solubility coefficient, and pK’ is the apparent pK.

S and pK’ were calculated as follow [2]:

S = 0.0307 + [0.00057 × (37 – T)] + [0.00002 × (37 – T)^2^]

and

pK’ = 6.086 + [0.042 × (7.4 – pH)] + ((38 – T) × {0.00472 + [0.00139 × (7.4 – pH)]})

where T is the temperature expressed as ^0^C.

**Study protocol**

At baseline, a first set of measurements was performed, including hemodynamic and tissue oxygenation variables (heart rate, mean arterial pressure, CI, DO_2_, VO_2_, ScvO_2_), arterial lactate level, ∆PCO_2_, ∆ContCO_2_/∆ContO_2_ ratio, and ∆PCO_2_/∆ContO_2_ ratio. A-500 ml of colloid solution (4% Human serum albumin, Vialebex®; LFB) was infused to the patient over 15 minutes via a specific venous line. Immediately after volume expansion, a second set of measurement was recorded, including hemodynamic and tissue oxygenation variables (heart rate, mean arterial pressure, CI, DO_2_, VO_2_, ScvO_2_), arterial lactate level, ∆PCO_2_, ∆ContCO_2_/∆ContO_2_ ratio, and ∆PCO_2_/∆ContO_2_ ratio. Ventilation parameters and doses of norepinephrine and sedation drugs were kept constant during the fluid challenge.

**References**

1. Douglas AR, Jones NL, Reed JW (1988) Calculation of whole blood CO_2_ content. J Appl Physiol 65:473-477
2. Austin WH, Lacombe E, Rand PW, Chatterjee M (1963) Solubility of carbon dioxide in serum from 15 to 38 C. J Appl Physiol 18:301-304
