# Supplementary material for: Ratios of central venous-to-arterial carbon dioxide content or tension to arteriovenous oxygen content are better markers of global anaerobic metabolism than lactate in septic shock patients
Source: Ann Intensive Care. 2016 Feb 3;6:10. doi: 10.1186/s13613-016-0110-3 (PMC4740480; doi:10.1186/s13613-016-0110-3)
Supplement: Supplementary file 2 — 10.1186/s13613-016-0110-3 Hemodynamic and tissue oxygenation parameters before and after 500 mL of volume expansion in responders and non-responders. [file 13613_2016_110_MOESM2_ESM.doc]

**Table S1.** Hemodynamic and tissue oxygenation parameters before and after 500 ml of volume expansion in Responders and Non-responders

|  | **Fluid-responders(n=51)** | | **Fluid-non-responders (n=47)** | |
| --- | --- | --- | --- | --- |
|  | **Before volume expansion** | **After volume expansion** | **Before volume expansion** | **After volume expansion** |
| **Heart rate, beats/min** | **109 ± 31** | **105 ± 26^*^** | **96 ± 20^#^** | **95 ± 20^#^** |
| **Mean arterial pressure, mmHg** | **69 ± 15** | **82 ± 12^*^** | **72 ± 13** | **81 ± 15^*^** |
| **Cardiac index, L/min/m^2^** | **2.6 [1.9-3.2]** | **3.5 [2.5-4.1]^*^** | **2.9 [2.5-3.6]** | **2.9 [2.5-4.0]** |
| **Stroke index, mL/m^2^** | **25.6 [16.0-34.1]** | **33.0 [26.1-41.4]^*^** | **32.4 [26.8-39.3]^#^** | **32.2 [28.6-41.6]** |
| **Arterial pH** | **7.32 [7.23-7.36]** | **7.35 [7.22-7.37]** | **7.38 [7.28-7.40]** | **7.36 [7.26-7.37]^*^** |
| **SaO_2_, %** | **96 [91-99]** | **97 [94-98]^*^** | **96 [94-98]** | **96 [94-98]** |
| **Hemoglobin, g/dL** | **10.4 ± 1.2** | **9.8 ± 1.0^*^** | **9.3 ± 1.2^#^** | **8.8 ± 1.0^#*^** |
| **CaO_2_, mL** | **12.9 ± 1.7** | **13.0 ± 1.5** | **11.6 ± 1.7^#^** | **11.5 ± 1.4^#^** |
| **PaCO_2_** | **35 [32-40]** | **35 [31-42]** | **38 [32-40]** | **39 [31-42]** |
| **Venous pH** | **7.24 [7.22-7.31]** | **7.27 [7.21-7.33]^*^** | **7.32 [7.24-7.36]** | **7.30 [7.24-7.34]^*^** |
| **ScvO_2_, %** | **61 ± 12** | **65 ± 10^*^** | **60 ± 15** | **62 ± 16** |
| **CcvO_2_, mL** | **8.3 ± 2.0** | **8.7 ± 1.8^*^** | **7.5 ± 2.5** | **7.6 ± 2.5^#^** |
| **PcvCO_2_, mmHg** | **45 [37-49]** | **44 [35-46]^*^** | **44 [40-49]** | **44 [40-48]** |
| **DO_2_, mL/min/m^2^** | **345 ± 100** | **445 ± 111^*^** | **368 ± 149** | **367 ± 136** |
| **VO_2_, mL/min/m^2^** | **118 ± 44** | **139 ± 33^*^** | **124 ± 46** | **119 ± 53^#^** |
| **OE** | **0.35 ± 0.12** | **0.33 ± 0.10^*^** | **0.36 ± 0.15** | **0.35 ± 0.16** |
| **∆PCO_2_, mmHg** | **7.0 [4.0-10.0]** | **5.0 [3.0-6.0]^*^** | **6.0 [4.0-8.0]** | **6.0 [5.0-7.0]** |
| **CcvCO_2_, mL** | **43.4 [40.2-50.1]** | **41.4 [36.3-47.0]^*^** | **51.0 [40.4-54.7]** | **48.3 [34.6-51.3]^#*^** |
| **CaCO_2_, mL** | **39.1 [35.6-48.5]** | **37.2 [34.6-43.6]^*^** | **50.2 [34.7-52.2]^#^** | **45.2 [32.6-48.3]^#*^** |
| **∆ContO_2_, mL** | **4.1 [3.5-6.0]** | **4.5 [3.6-5.3]** | **4.5 [3.2-4.8]** | **3.5 [3.0-4.3]** |
| **∆PCO_2_/∆ContO_2_, mmHg/mL** | **1.68 [1.05-2.33]** | **1.20 [1.05-1.67]^*^** | **1.60 [1.03-2.07]** | **1.48 [1.37-1.80]^#^** |
| **∆ContCO_2_, mL** | **4.14 [1.81-5.20]** | **2.96 [1.10-3.37]^*^** | **2.80 [1.41-3.27]^#^** | **2.46 [2.20-3.18]** |
| **∆ContCO_2_/∆ContO_2_** | **1.00 [0.53-1.26]** | **0.65 [0.34-0.70]^*^** | **0.67 [0.38-0.83]^#^** | **0.70 [0.50-0.88]** |
| **Lactate, mmol/L** | **4.6 [2.5-6.3]** | **4.1 [2.0-5.8]^*^** | **2.1 [1.4-4.4]^#^** | **1.7 [1.3-4.0]^#*^** |

SaO_2_: arterial oxygen saturation; CaO_2_: arterial oxygen content; PaCO_2_: arterial carbon dioxide tension; ScvO_2_: central venous oxygen saturation; CcvO_2_: central venous oxygen content; PcvCO_2_: central venous carbon dioxide tension; DO_2_: oxygen delivery; VO_2_: oxygen consumption; ∆PCO_2_: venous-arterial carbon dioxide tension difference; CaCO_2_, arterial carbon dioxide content; CcvCO_2_, central venous carbon dioxide content; ∆ContO_2_, arterial-to-central venous oxygen content difference; ∆ContCO_2_, central venous-to-arterial carbon dioxide content difference; OE, oxygen extraction. Data are expressed as mean ± SD or as median [interquartile range, 25-75].

^*^, p <0.05 after vs. before volume expansion. ^#^, p <0.05 patients with fluid-responders vs. patients with fluid-non-responders.
