# Supplementary material for: Ratios of central venous-to-arterial carbon dioxide content or tension to arteriovenous oxygen content are better markers of global anaerobic metabolism than lactate in septic shock patients
Source: Ann Intensive Care. 2016 Feb 3;6:10. doi: 10.1186/s13613-016-0110-3 (PMC4740480; doi:10.1186/s13613-016-0110-3)
Supplement: Supplementary file 3 — 10.1186/s13613-016-0110-3 Hemodynamic and tissue oxygenation parameters before and after 500 mL of volume expansion according to the baseline ScvO2 groups. [file 13613_2016_110_MOESM3_ESM.doc]

|  | **Baseline ScvO_2_ >70% (n=28)** | | **Baseline ScvO_2_ ≤70% (n=70)** | |
| --- | --- | --- | --- | --- |
|  | **Before volume expansion** | **After volume expansion** | **Before volume expansion** | **After volume expansion** |
| **Heart rate, beats/min** | **87 ± 18** | **90 ± 18** | **109 ± 27^#^** | **105 ± 24^#^*** |
| **Mean arterial pressure, mmHg** | **72 ± 15** | **87 ± 10^*^** | **70 ± 14** | **79 ± 14^#*^** |
| **Cardiac index, L/min/m^2^** | **2.59 [2.47-3.54]** | **3.02 [2.76-4.08]^*^** | **2.75 [2.10-3.28]** | **3.30 [2.27-3.91]^*^** |
| **Stroke index, mL/m^2^** | **35.9 [31.0-40.0]** | **41.4 [34.3-49.3]^*^** | **26.8 [19.4-32.6]^#^** | **31.6 [26.1-38.7]^#*^** |
| **Arterial pH** | **7.36 [7.29-7.42]** | **7.36 [7.27-7.38]** | **7.32 [7.21-7.38]^#^** | **7.35 [7.22-7.36]^#*^** |
| **SaO_2_, %** | **97 [94-99]** | **97 [95-98]** | **96 [93-98]** | **96 [94-98]^#^** |
| **Hemoglobin, g/dL** | **10.5 ± 1.2** | **10.0 ± 1.2^*^** | **9.6 ± 1.3^#^** | **9.1 ± 1.0^#*^** |
| **CaO_2_, mL** | **13.2 ± 1.6** | **13.3 ± 1.4** | **11.9 ± 1.8^#^** | **11.9 ± 1.5^#^** |
| **PaCO_2_** | **39 ± 8** | **39 ± 8** | **36 ± 7** | **36 ± 7** |
| **CaCO_2_, mL** | **50.2 [46.9-52.2]** | **46.9 [42.4-48.1]^*^** | **37.1 [31.4-49.0]^#^** | **37.2 [28.8-43.6]^#*^** |
| **Venous pH** | **7.33 [7.25-7.40]** | **7.33 [7.25-7.36]^*^** | **7.28 [7.18-7.33]^#^** | **7.27 [7.20-7.33]^#*^** |
| **ScvO_2_, %** | **76 ± 4** | **76 ± 7** | **54 ± 10^#^** | **59 ± 12^#*^** |
| **CcvO_2_, mL** | **10.6 ± 1.3** | **10.5 ± 1.5** | **6.9 ± 1.6^#^** | **7.3 ± 1.8^#*^** |
| **PcvCO_2_, mmHg** | **44 ± 8** | **44 ± 8** | **44 ± 7** | **42 ± 7^*^** |
| **CcvCO_2_, mL** | **53.3 [49.5-53.7]** | **49.8 [44.9-51.3]^*^** | **43.4 [35.7-51.0]^#^** | **41.3 [31.8-47.3]^#*^** |
| **DO_2_, mL/min/m^2^** | **413 ± 155** | **475 ± 147^*^** | **333 ± 105^#^** | **382 ± 112^#*^** |
| **VO_2_, mL/min/m^2^** | **81 ± 26** | **97 ± 24^*^** | **137 ± 41^#^** | **142 ± 44^#^** |
| **OE** | **0.20 ± 0.04** | **0.21 ± 0.05** | **0.42 ± 0.10^#^** | **0.38 ± 0.12^#*^** |
| **∆PCO_2_, mmHg** | **5.0 [3.0-6.0]** | **5.0 [3.0-6.0]** | **7.0 [4.0-10.0]^#^** | **5.5 [5.0-8.0]^*^** |
| **∆ContO_2_, mL** | **2.73 [2.44-3.20]** | **2.79 [2.25-3.54]** | **4.62 [4.03-6.00]^#^** | **4.38 [3.58-5.80]^#*^** |
| **∆PCO_2_/∆ContO_2_, mmHg/mL** | **2.07 [1.05-2.20]** | **1.78 [1.40-1.98]^*^** | **1.43 [1.03-2.00]** | **1.30 [1.09-1.64]^#*^** |
| **∆ContCO_2_, mL** | **2.60 [1.15-3.04]** | **2.46 [1.43-3.22]** | **4.03 [2.17-5.20]^#^** | **2.62 [1.92-3.33]^*^** |
| **∆ContCO_2_/∆ContO_2_** | **0.94 [0.56-1.32]** | **0.88 [0.68-0.93]** | **0.69 [0.41-1.05]** | **0.65 [0.39-0.70]^#*^** |
| **Lactate, mmol/L** | **2.45 [1.40-5.60]** | **2.00 [1.30-4.40]^*^** | **4.10 [1.50-6.30]** | **3.70 [1.70-5.20]^*^** |

**Table S2.** Hemodynamic and tissue oxygenation parameters before and after 500 ml of volume expansion according to the baseline ScvO_2_ groups

SaO_2_: arterial oxygen saturation; CaO_2_: arterial oxygen content; PaCO_2_: arterial carbon dioxide tension; ScvO_2_: central venous oxygen saturation; CcvO_2_: central venous oxygen content; PcvCO_2_: central venous carbon dioxide tension; DO_2_: oxygen delivery; VO_2_: oxygen consumption; ∆PCO_2_: venous-arterial carbon dioxide tension difference; CaCO_2_, arterial carbon dioxide content; CcvCO_2_, central venous carbon dioxide content; ∆ContO_2_, arterial-to-central venous oxygen content difference; ∆ContCO_2_, central venous-to-arterial carbon dioxide content difference; OE, oxygen extraction. Data are expressed as mean ± SD or as median [interquartile range, 25-75].

^*^, p <0.05 after vs. before volume expansion. ^#^, p <0.05 patients with a ScvO_2_ ≤70% vs. patients with a ScvO_2_ <70%.
